# Minimization of border effects in monolithic scintillators using neural networks, based on MR-compatible SiPM arrays

**DOI:** 10.1186/2197-7364-1-S1-A19

**Published:** 2014-07-29

**Authors:** Pablo Conde, Amadeo Iborra, Antonio J González, Liczandro Hernández, Pablo Bellido, Efrén Crespo, Laura Moliner, Juan P Rigla, María J Rodríguez-Álvarez, Filomeno Sánchez, Michael Seimetz, Antonio Soriano, Luis Fernando Vidal, José M Benlloch

**Affiliations:** Institute for instrumentation in Molecular Imaging, CSIC-UPV, Valencia, Spain

Neural Networks (NNs) have been suggested to fit experimental data in an efficient way when compared to other methods such as least squares fitting. The advantage of NNs is the avoidance of iterative processes and that it does not require initialization parameters. Furthermore, it is possible to implement the network in special purpose hardware, as FPGAs, thereby exploiting the intrinsically parallel nature of NNs.

Two detector modules in coincidence were used for the test measurements. Each of them was composed of an array of 144 SiPMs and a monolithic LYSO crystal covering the whole array area. The scintillator slab had all faces black painted except the one in contact with the photodetectors. Each row and column of the photodetectors array (12+12) is digitized using a diode network in order to obtain the projections of the light distribution of every event.

In order to minimize the SiPMs dark noise contribution to the measures, both modules were kept at a constant temperature (17ºC). The modules are placed face-to-face at a fixed distance on top of a cold plate to which a chiller system pumps liquid refrigerant.

Two identical NNs composed of twelve inputs, one hidden layer and twelve sigmoid shaped neurons were trained using the Levenberg-Marquardt algorithm with simulated data sets.

Preliminary tests have shown the capability of using NNs to reconstruct the impinging photons map within the scintillation crystal using a monolithic crystal and rows and columns from a SiPM array as data input. The border effects when the CoG approach is used can be minimized with the NN fitting approach. We have faced a training process of the neural network that showed to be a critical issue in order to obtain a correct estimation of the parameters of the light distribution. Further explorations in training algorithms will be made.

